# An extensively validated whole-cell biosensor for specific, sensitive and high-throughput detection of antibacterial inhibitors targeting cell-wall biosynthesis

**DOI:** 10.1093/jac/dkac429

**Published:** 2023-01-10

**Authors:** Luiza H Galarion, Jennifer K Mitchell, Christopher P Randall, Alex J O’Neill

**Affiliations:** School of Molecular and Cellular Biology, Faculty of Biological Sciences, University of Leeds, Leeds LS2 9JT, UK; School of Molecular and Cellular Biology, Faculty of Biological Sciences, University of Leeds, Leeds LS2 9JT, UK; School of Molecular and Cellular Biology, Faculty of Biological Sciences, University of Leeds, Leeds LS2 9JT, UK; School of Molecular and Cellular Biology, Faculty of Biological Sciences, University of Leeds, Leeds LS2 9JT, UK

## Abstract

**Background:**

Whole-cell biosensor strains are powerful tools for antibacterial drug discovery, in principle allowing the identification of inhibitors acting on specific, high-value target pathways. Whilst a variety of biosensors have been described for detecting cell-wall biosynthesis inhibitors (CWBIs), these strains typically lack specificity and/or sensitivity, and have for the most part not been rigorously evaluated as primary screening tools. Here, we describe several *Staphylococcus aureus* CWBI biosensors and show that specific and sensitive biosensor-based discovery of CWBIs is achievable.

**Methods:**

Biosensors comprised *lacZ* reporter fusions with *S. aureus* promoters (P*_gltB_*, P*_ilvD_*, P*_murZ_*, P*_oppB_*, P*_ORF2768_*, P*_sgtB_*) that are subject to up-regulation following inhibition of cell-wall biosynthesis. Induction of biosensors was detected by measuring expression of β-galactosidase using fluorogenic or luminogenic substrates.

**Results:**

Three of the six biosensors tested (those based on P*_gltB_*, P*_murZ_*, P*_sgtB_*) exhibited apparently specific induction of β-galactosidase expression in the presence of CWBIs. Further validation of one of these (P*_murZ_*) using an extensive array of positive and negative control compounds and conditional mutants established that it responded appropriately and uniquely to inhibition of cell-wall biosynthesis. Using this biosensor, we established, validated and deployed a high-throughput assay that identified a potentially novel CWBI from a screen of >9000 natural product extracts.

**Conclusions:**

Our extensively validated P*_murZ_* biosensor strain offers specific and sensitive detection of CWBIs, and is well-suited for high-throughput screening; it therefore represents a valuable tool for antibacterial drug discovery.

## Introduction

Nearly a century on from discovery of the first antibiotic capable of inhibiting bacterial cell-wall biosynthesis (penicillin), the pathway by which the peptidoglycan cell wall is constructed remains amongst the most attractive targets for antibacterial drug discovery.^1^ There are several reasons for this above and beyond the simple fact that cell-wall biosynthesis inhibitors (CWBIs) have proven to be a rich source of antibacterial drugs to date.

First, the cell wall is an essential feature of most bacterial pathogens, and its structure and biosynthesis are generally well conserved, thereby offering the opportunity to identify antibacterial inhibitors exhibiting broad-spectrum activity. Second, there is no comparable structure or biosynthetic pathway in mammalian cells, which avoids the spectre of mechanism-based toxicity for novel CWBIs and increases the likelihood that such compounds will exhibit profound selectivity against bacteria. Third, the final steps of cell-wall biosynthesis occur on the outer surface of the cytoplasmic membrane, and are—at least in Gram-positive bacteria—readily accessible to CWBIs; given that achieving effective delivery of small-molecule inhibitors into bacteria remains a prominent challenge in antibacterial discovery,^2^ this is undoubtedly a desirable feature of the pathway. Finally, some CWBI targets are associated with a substantially lower potential for resistance through mutation than is observed for other bacterial drug targets.^2,3^

In view of the benefits of cell-wall biosynthesis as a target pathway for antibacterial drugs, there exists a long history of screening approaches for targeted discovery of CWBIs. Indeed, methods to specifically identify CWBIs over other types of antibacterial inhibitor were already in use within the pharmaceutical industry as primary screens by the early 1960s.^4^ Amongst the most productive of such screening approaches have been the spheroplasting assay, in which the presence of a CWBI induces the formation of refractile spheroplasts from bacteria growing in osmotically buffered medium, and the L-form assay, which exploits the fact that bacteria devoid of peptidoglycan (‘L-forms’) become differentially resistant to CWBIs relative to their walled counterparts.^4,5^ These assays have proven extremely fruitful in the past, collectively underpinning the discovery of several important CWBIs, including the carbapenems (thienamycin), fosfomycin, ramoplanin and teicoplanin.^4^

In recent years, attempts to establish next-generation CWBI-specific assays with improved performance and better suited for high-throughput screening applications have had a major focus on whole-cell biosensor strains. Biosensors in this context are bacteria containing reporter genes fused to promoters responsive to antibiotic-induced stress, and in principle offer a powerful approach for identification of antibacterial inhibitors acting on specific, high-value target pathways such as cell-wall biosynthesis.^6,7^ In practice, existing biosensors for CWBI detection suffer from a number of drawbacks, and the technology has in our view yet to fulfil its considerable potential in this setting. Of the reported biosensors capable of detecting CWBIs, a near-universal theme is that—in addition to showing induction of reporter gene expression in response to CWBIs—they also exhibit induction in response to membrane-active agents (Table 1).^7–17^

**Table 1.** Overview of previously described whole-cell biosensors responsive to inhibitors of cell-wall biosynthesis

Host species	Promoter	Potential limitation(s)	References
**Gram-negative**			
*ȃEscherichia coli*	*ampC*	induced by membrane-active compounds, including detergents and inhibitors of outer membrane biogenesis	^8,10^
	P3*rpoH*	induced by membrane-active compounds (polymyxin B)	^11^
**Gram-positive**			
*ȃBacillus subtilis*	*liaI*	induced by membrane-active compounds, including surfactant (BDMHDA-Cl) and organic solvents (diphenyl ether, n-hexane, cyclooctane)	^12^
	*vanH*	induced by membrane-active compounds, including detergents and surfactants (TX-100, NP-40 and SDS); not induced by the CWBI D-cycloserine	^13^
	*ypbG*	induced by membrane-active compounds (polymyxin B); fails to respond to some CWBIs (ristocetin); Z′ factor is <0, indicating lack of suitability for high-throughput screening	^15^
	*ypuA*	induced by membrane-active compounds (polymyxin B, nisin)	^14^
	*ywaC*	induced by membrane-active compounds (polymyxin B, EDTA)	^7^
*ȃEnterococcus faecalis*	*vanH*	induced by non-CWBIs trimethoprim, sulfamethoxazole and daptomycin; not induced by CWBIs D-cycloserine, fosfomycin, and some β-lactams	^16,17^
	
*ȃStaphylococcus aureus*	*pbp2*, *tcaA*, *vraSR*, *sgtB*, *lytR*	limited validation in respect of specificity and sensitivity	^9^

The latter are typically considered nuisance compounds in antibacterial discovery,^3^ and are prevalent in synthetic compound libraries of the type often employed in modern drug discovery campaigns;^18^ thus, the failure of most CWBI biosensors to discriminate between compounds that target the cell wall and those that hit the bacterial envelope is a significant limitation. This lack of specificity in CWBI biosensors is not infrequently accompanied by a lack of sensitivity, i.e. a failure to respond universally to known CWBIs (Table 1),^9,13,15–17^ which implies that such biosensors will be liable to miss novel CWBIs when used in the context of a primary discovery screen. Further limiting their application in antibacterial drug discovery is the fact that, whilst existing CWBI biosensors have generally undergone a level of validation sufficient to qualify them for use as research tools, their performance in the context of a primary screen for CWBIs has in many cases yet to be established.

In a previous study, we described the generation of a *Staphylococcus aureus* biosensor that employed the *murZ* promoter to report on inhibition of cell-wall biosynthesis.^19^ Although tested with only a handful of antibiotic classes, this biosensor appeared to respond appropriately to CWBIs. Unfortunately, it also exhibited an apparent lack of specificity, in that it responded to a non-CWBI compound, the transcription inhibitor rifampicin. The present studies were initiated to generate staphylococcal CWBI biosensors with improved specificity, and to explore their potential for use as a screening tool to identify novel CWBIs. Here we describe the validation of *S. aureus* biosensor strains with an apparently unrivalled level of sensitivity and specificity amongst those reported in the literature, and establish and deploy a high-throughput biosensor assay for successful detection of CWBIs.

## Material and methods

### Whole-cell biosensor strains

Existing transcriptional profiling data for *S. aureus* were interrogated to identify genes uniquely subject to up-regulation following inhibition of cell-wall biosynthesis;^20,21^ five were selected for the generation of biosensor constructs (see Results section). For each of these, a DNA fragment encompassing the promoter was amplified by PCR using the oligonucleotides listed in Table S1, available as Supplementary data at *JAC* Online. The amplicons were ligated into a modified version of plasmid pAD123,^22^ in which the *gfp* gene was replaced with *lacZ* from pMUTIN4.^23^ The resulting transcriptional fusion constructs were established in *E. coli* DH5α and verified by DNA sequencing, before electroporation^24^ into *S. aureus* RN4220.^25^ A biosensor that carries a chromosomal *murZ*::*lacZ* fusion (referred to hereafter as the P*_murZ_* biosensor), was previously generated in our laboratory.^19^ To examine the response of this latter biosensor construct in strains of *S. aureus* RN4220 carrying conditional [temperature-sensitive (Ts)] mutations in cell-wall biosynthesis proteins (GlmM, MurC, MurF, FmhB)^26^ or an unrelated protein (DnaA),^27^ the *murZ*::*lacZ* fusion was transduced into these strains using bacteriophage Φ11.^28^

### Initial biosensor assay

CWBIs and negative control compounds were either from Sigma–Aldrich or the sources listed in Table S2. Susceptibility testing^29^ was used to define appropriate concentrations of control compounds for assay. Biosensor strains were cultured in tryptone soya broth (TSB; Oxoid) at 37°C with vigorous aeration to an OD_600_ of 0.2 and challenged with antimicrobial agents for 60 min. In the case of biosensor constructs in Ts mutants, strains were grown at 30°C to an OD_600_ of 0.2, before shifting the temperature to 42°C for 60 min. Post-challenge, OD_600_ was measured to allow changes in cell density to be accounted for in calculating biosensor induction. An aliquot of culture (typically 200 µL) was centrifuged, and the washed cells resuspended in 0.5 volumes of AB buffer^30^ containing lysostaphin (15 mg/L) and the fluorogenic β-galactosidase (β-gal) substrate, 4-methylumbelliferyl β-D-galactopyranoside (MUG, 500 mg/L; Sigma–Aldrich), and incubated at 25°C with shaking for 90 min. Production of β-gal was determined as described.^30^

### Biosensor assay for screening activities

For screening of the National Institutes of Health (NIH) Clinical Collection, a subset of the MicroSource Spectrum library (MicroSource Discovery Systems) and the Tocriscreen Total library (Tocris), compounds were dissolved in DMSO and tested in 96-well microtitre plates at a final well concentration of 10 µM. Detection of β-gal utilised the Beta-Glo^®^ assay system (Promega), as outlined below.

The P*_murZ_* biosensor assay was subsequently miniaturized for high-throughput screening in 384-well plate format using a total well volume of 50 µL. To prepare the biosensor, a 1/100 dilution of a saturated culture was grown in TSB at 37°C to an OD_600_ of 0.2. The biosensor (45 µL per well) was challenged with 5 µL of test compound in a clear F-bottom plate (Greiner Bio-One). A total of 9328 natural product extracts from the National Cancer Institute (NCI) Natural Products Open Repository Program^31^ were dissolved in DMSO at a stock concentration of 2 mg/mL, and tested at a final well concentration of 200 mg/L. Each screening plate included both positive and negative controls (10 µM penicillin and 10 µM tetracycline, respectively). Post-challenge, OD_600_ was measured to allow changes in cell density to be accounted for in calculating β-gal expression. The culture was mixed in a 9:1 ratio with Beta-Glo reagent in a LUMITRAC plate (Greiner Bio-One) by shaking for 45 s, and incubated in the dark at 25°C for 60 min before measuring luminescence. Induction was detected by comparing β-gal production per OD_600_ unit against the untreated control (biosensor culture in the presence of 10% DMSO).

### Further evaluation of hits identified using the P_murZ_ biosensor

Putative CWBIs from the NCI screen were tested for their ability to induce spheroplast formation in *Bacillus subtilis* ATCC 39374. Cells were prepared for spheroplasting as described.^32^ Test extracts were added to cells and incubated for 3 h, with hourly removal of aliquots for observation by microscopy. Penicillin (3 ng/mL) and lysozyme (5 mg/mL) were used as positive controls for spheroplasting, with tetracycline (250 ng/mL) employed as a negative control.

To assess whether hit extracts contained known CWBIs, tandem MS data were obtained by running extract through a C18 column on a Dionex 3000RS UHPLC coupled to a Bruker Ultra High Resolution Q-TOF maXis mass spectrometer with an electrospray source operating in positive-ion mode and scanning of *m/z* from 50 to 2000. The resulting spectral data were uploaded to the Global Natural Products Social Molecular Networking (GNPS: https://gnps.ucsd.edu/ProteoSAFe/static/gnps-splash.jsp) site and analysed using the Spectral Library Search workflow. To detect the presence of peptides with *m/z *> 2000, protein LC-MS/MS was performed using existing methods for sample clean-up^33^ and analysis.^34^

## Results and discussion

### Generation and initial evaluation of biosensors

With the aid of existing transcriptional profiling data for *S. aureus* challenged with CWBIs^20,21^ or a variety of antibacterial compounds with other cellular targets,^21^ we selected five genes apparently uniquely subject to up-regulation in the former case as candidates for generating CWBI-responsive biosensors: *gltB* (SAOUHSC_00435), *oppB* (SAOUHSC_00923), *sgtB* (SAOUHSC_02012), *ilvD* (SAOUHSC_02281) and *ORF2768* (SAOUHSC_03021). To our knowledge, only one of these (*sgtB*) has previously been employed as the basis for a staphylococcal CWBI biosensor.^9^ For each of these, we generated transcriptional fusions with the *lacZ* gene, thereby placing β-gal production under their control. The resulting five biosensor constructs were evaluated alongside the previously generated P*_murZ_* biosensor for their response to a small collection of CWBIs and several antibacterial agents that do not target cell-wall biosynthesis. In line with an established fold change considered significant for gene expression analysis, we initially chose a 2-fold increase in β-gal production as a threshold to define successful induction.

Three of the six constructs reliably exhibited ≥2-fold induction of β-gal in the presence of CWBIs; P*_gltB_*, P*_sgtB_* and P*_murZ_* (Table S3). All three also failed to show induction above the threshold in the presence of antibacterial agents that target other cellular processes, including rifampicin (Table S3). This latter finding came as a surprise for the P*_murZ_* biosensor, since we had consistently detected induction following challenge with rifampicin in our previous study.^19^ Nevertheless, repeated checking here using multiple independent batches of rifampicin and the closely related rifamycin SV, established that the earlier result was an artefact, and that the P*_murZ_* biosensor is not induced by this class. Testing of a larger collection of positive and negative control agents against the three biosensors suggested that they are all uniquely responsive to CWBIs (data not shown). On the basis that the P*_murZ_* construct typically showed the greatest induction in response to CWBI challenge, we elected to move forward to more extensive validation and application with this biosensor alone.

### Assessment of the sensitivity and specificity of the P_murZ_ biosensor

**Figure 1.**
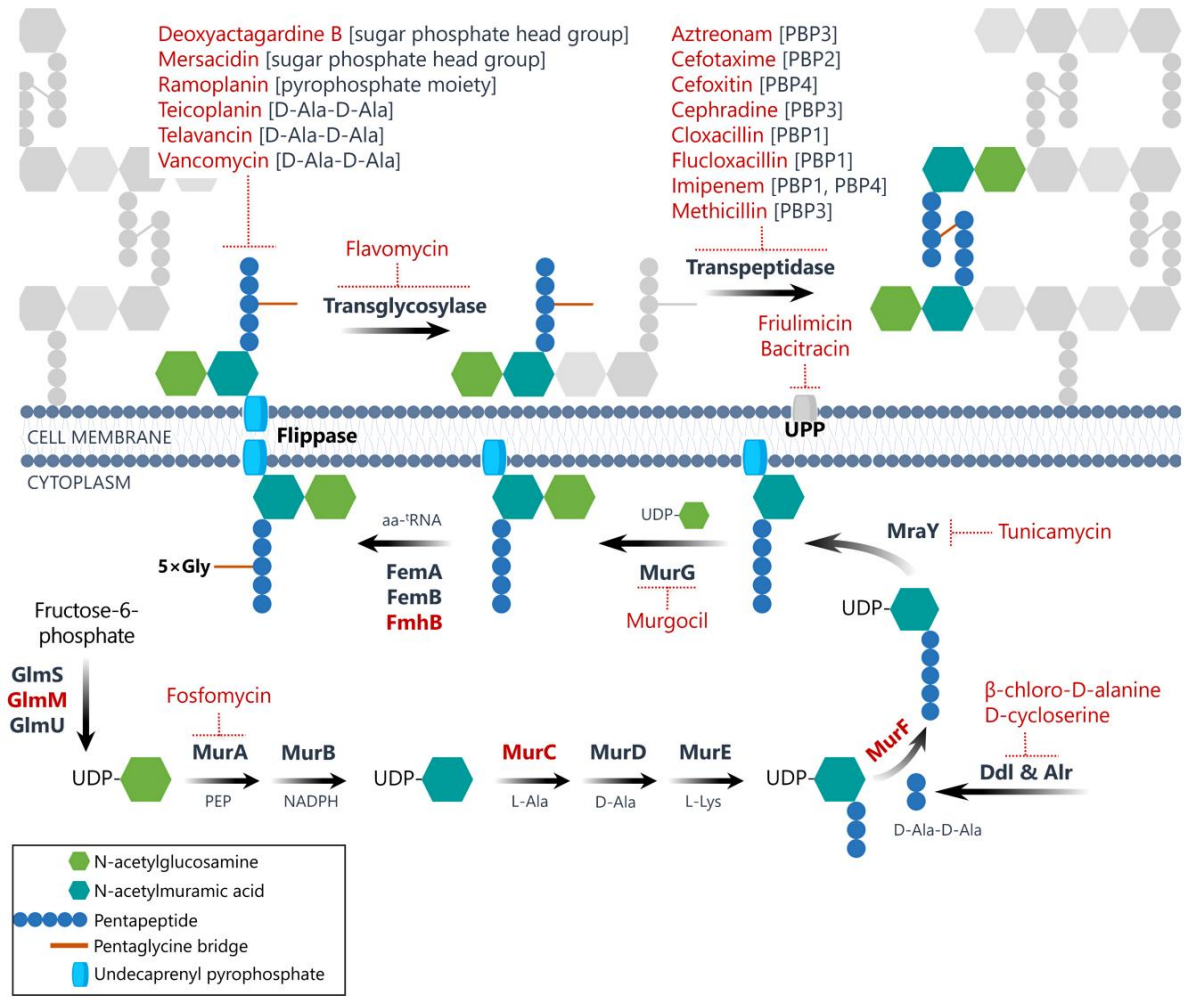
Cell-wall biosynthesis in *S. aureus*, and the individual targets and inhibitors of this pathway used in this study for biosensor validation. Enzymes targeted through use of thermosensitive mutants are shown in red boldface type, whilst chemical inhibitors are in red, non-boldface type. The specific targets of individual lipid II binders and the primary targets of transpeptidase inhibitors are indicated in brackets.^35–45^ Target information for other CWBIs derives from several sources.^46–52^

Table 2 shows the response of the P*_murZ_* biosensor to a broad cross-section of known CWBIs (Figure 1). Challenge with any of these agents resulted in ≥2-fold increase in β-gal expression at or above the corresponding MIC of the compound in all cases, and in several instances induction was also observed at a subinhibitory concentration (0.25× MIC) (Table 2).

**Table 2.** The P_murZ_ biosensor shows induction of β-gal expression (values in bold) at or above the threshold (2-fold) in the presence of all cell-wall active agents tested

Antibacterial agent	MIC (mg/L)	Fold induction (± SD)		
		0.25× MIC	1× MIC	4× MIC
Inhibitors of intracellular steps of cell-wall biosynthesis				
*ȃ*β-Chloro-ᴅ-alanine	2048	1.9 ± 0.3	**2.4 **±** 0.3**	**8.2 **±** 1.9**
*ȃ*ᴅ-cycloserine	64	1.0 ± 0.5	**2.8 **±** 0.2**	**3.7 **±** 0.7**
*ȃ*Fosfomycin	8	1.9 ± 0.1	**3.1 **±** 0.4**	**4.6 **±** 0.9**
*ȃ*Murgocil	8	1.4 ± 0.1	**2.6 **±** 0.4**	**3.0 **±** 0.3**
*ȃ*Tunicamycin	8	1.9 ± 0.2	**2.6 **±** 0.1**	**2.9 **±** 0.4**
Inhibitors of extracellular steps of cell-wall biosynthesis				
*ȃ*Aztreonam	512	1.2 ± 0.2	**5.6 **±** 1.4**	**5.2 **±** 1.1**
*ȃ*Bacitracin	128	**5.5 **±** 0.4**	**6.7 **±** 0.7**	**5.3 **±** 0.3**
*ȃ*Cefotaxime	2	**6.8 **±** 0.7**	**7.6 **±** 0.2**	**6.3 **±** 0.7**
*ȃ*Cefoxitin	4	1.4 ± 0.1	**5.5 **±** 0.6**	**7.3 **±** 0.4**
*ȃ*Cefradine	8	1.0 ± 0.1	**2.0 **±** 0.0**	**5.1 **±** 1.2**
*ȃ*Cloxacillin	0.062	1.0 ± 0.0	**2.2 **±** 0.0**	**3.9 **±** 0.5**
*ȃ*Deoxyactagardine B	32	1.6 ± 0.1	**3.2 **±** 0.5**	**2.1 **±** 0.2**
*ȃ*Flavomycin	4	1.2 ± 0.1	**4.2 **±** 0.4**	**3.9 **±** 0.3**
*ȃ*Flucloxacillin	0.25	1.6 ± 0.1	**7.2 **±** 0.9**	**7.8 **±** 1.9**
*ȃ*Friulimicin	4	1.2 ± 0.3	**2.7 **±** 0.9**	**4.8 **±** 0.7**
*ȃ*Imipenem	0.062	1.3 ± 0.0	**2.9 **±** 0.4**	**4.6 **±** 0.3**
*ȃ*Mersacidin	32	**2.4 **±** 0.3**	**3.7 **±** 0.8**	**2.9 **±** 0.3**
*ȃ*Methicillin	4	1.4 ± 0.0	**4.6 **±** 0.1**	**10.4 **±** 0.1**
*ȃ*Penicillin G	0.031	0.7 ± 0.1	**3.1 **±** 0.1**	**6.2 **±** 0.7**
*ȃ*Ramoplanin	2	1.6 ± 0.3	**2.3 **±** 0.6**	**2.3 **±** 0.2**
*ȃ*Teicoplanin	4	**3.8 **±** 0.8**	**3.2 **±** 0.9**	**2.8 **±** 0.5**
*ȃ*Telavancin	1	**4.6 **±** 0.8**	**4.5 **±** 0.4**	**4.3 **±** 0.5**
*ȃ*Vancomycin	2	0.7 ± 0.1	**3.7 **±** 0.4**	**3.8 **±** 0.5**

Data generated using MUG as the β-gal substrate.

Whilst the compounds tested included all of the known CWBIs that we were able to source, and encompassed a broad range of processes that form part of cell-well biosynthesis (Figure 1), we additionally sought to establish that the P*_murZ_* biosensor would respond to inhibition of other targets in the pathway for which appropriate chemical inhibitors were not available. To achieve this, we introduced the P_*murZ*_ reporter construct into staphylococcal Ts mutants of proteins involved in precursor supply for cell-wall biosynthesis (the phosphoglucosamine mutase, GlmM), assembly of the pentapeptide moiety of peptidoglycan monomers (amide ligases, MurC and MurF) and pentaglycine bridge formation (the aminoacyl transferase, FmhB) (Figure 1). In all cases, growing these strains at the non-permissive temperature to inhibit protein function resulted in successful induction of β-gal expression (GlmM: 3.1 ± 0.1, MurC: 3.5 ± 0.2, MurF: 3.0 ± 0.1, FmhB: 2.5 ± 0.2). By contrast, a Ts variant of a target unrelated to cell-wall biosynthesis (DNA replication initiator protein, DnaA) displayed no induction of P_*murZ*_ under these conditions (1.1 ± 0.2).

A broad array of negative control compounds was also tested to confirm that the biosensor was uniquely responsive to CWBIs; this included 32 antibacterial compounds acting on targets other than the cell wall (Table 3), of which 9 are membrane-active agents, and a representative selection of 13 pan-assay interference compounds (PAINs; so-called because they are a frequent source of false-positive signals in screens) (Table 4).^53,54^ All of these compounds failed to induce β-gal expression above the threshold.

**Table 3.** The P_murZ_ biosensor does not show induction at or above the threshold (2-fold) when challenged with inhibitors targeting cellular structures or processes unrelated to cell-wall biosynthesis

Antibacterial agent	MIC (mg/L)	Fold induction (± SD)		
		0.25× MIC	1× MIC	4× MIC
Membrane-active agents				
*ȃ*Anhydrotetracyline	2	1.3 ± 0.2	1.1 ± 0.0	1.1 ± 0.1
*ȃ*CCCP	2	1.1 ± 0.0	1.2 ± 0.2	1.6 ± 0.3
*ȃ*Chlorhexidine	1	1.4 ± 0.1	1.3 ± 0.1	1.1 ± 0.0
*ȃ*CTAB	2	0.5 ± 0.1	0.9 ± 0.3	0.2 ± 0.1
*ȃ*Daptomycin	2	1.0 ± 0.1	1.4 ± 0.1	1.1 ± 0.4
*ȃ*EDTA	16	1.1 ± 0.2	0.7 ± 0.2	0.7 ± 0.1
*ȃ*Nisin	4	1.3 ± 0.3	1.1 ± 0.2	0.8 ± 0.0
*ȃ*Polymyxin B	16	1.2 ± 0.2	1.2 ± 0.1	1.3 ± 0.0
*ȃ*Valinomycin	2	0.7 ± 0.1	0.7 ± 0.1	0.1 ± 0.1
DNA synthesis inhibitors				
*ȃ*Acriflavine	32	1.1 ± 0.1	1.0 ± 0.1	0.6 ± 0.0
*ȃ*Ciprofloxacin	1	0.4 ± 0.1	0.4 ± 0.1	0.3 ± 0.1
*ȃ*Gepotidacin	0.125	1.2 ± 0.4	0.9 ± 0.3	0.6 ± 0.2
*ȃ*Nalidixic acid	64	0.9 ± 0.1	1.1 ± 0.2	1.1 ± 0.2
*ȃ*Novobiocin	0.125	1.3 ± 0.4	1.2 ± 0.4	1.8 ± 0.1
RNA synthesis inhibitors				
*ȃ*Rifampicin	0.015	0.7 ± 0.3	0.5 ± 0.1	0.4 ± 0.1
*ȃ*Rifamycin SV	0.008	0.2 ± 0.0	1.1 ± 0.0	1.1 ± 0.0
Protein synthesis inhibitors				
*ȃ*Actinonin	4	0.8 ± 0.2	0.7 ± 0.2	0.8 ± 0.1
*ȃ*Clindamycin	0.125	0.8 ± 0.1	1.1 ± 0.0	1.1 ± 0.0
*ȃ*Fusidic acid	0.125	1.2 ± 0.1	1.1 ± 0.1	1.1 ± 0.1
*ȃ*Gentamicin	1	1.1 ± 0.2	1.1 ± 0.1	1.0 ± 0.1
*ȃ*Linezolid	1	1.2 ± 0.2	1.2 ± 0.1	1.0 ± 0.2
*ȃ*Mupirocin	0.062	1.2 ± 0.1	1.1 ± 0.2	1.1 ± 0.1
*ȃ*Spectinomycin	64	1.1 ± 0.3	1.0 ± 0.2	0.8 ± 0.1
*ȃ*Streptomycin	4	1.0 ± 0.0	0.9 ± 0.0	0.7 ± 0.1
*ȃ*Tetracycline	0.5	0.9 ± 0.1	0.6 ± 0.1	0.5 ± 0.1
*ȃ*Tiamulin	1	0.9 ± 0.0	0.9 ± 0.0	1.0 ± 0.1
*ȃ*Tigecycline	0.5	0.8 ± 0.0	0.8 ± 0.0	0.9 ± 0.1
*ȃ*Virginiamycin	4	0.9 ± 0.2	0.9 ± 0.1	0.9 ± 0.2
Folate synthesis inhibitors				
*ȃ*Sulfamethoxazole	64	0.9 ± 0.0	0.9 ± 0.1	0.5 ± 0.0
*ȃ*Trimethoprim	8	1.0 ± 0.1	1.1 ± 0.1	1.3 ± 0.1
Fatty acid synthesis inhibitors				
*ȃ*Batumin	0.25	0.0 ± 0.6	0.0 ± 0.5	0.0 ± 0.4
*ȃ*Triclosan	0.125	0.5 ± 0.1	0.4 ± 0.2	0.9 ± 0.1

CCCP, carbonyl cyanide *m*-chlorophenylhydrazone; CTAB, cetyltrimethylammonium bromide; EDTA, ethylenediaminetetraaceticacid. Data generated using MUG as the β-gal substrate.

**Table 4.** The P_murZ_ biosensor does not show induction at or above the threshold (2-fold) when challenged with a variety of pan-assay interference compounds (PAINs) at 100 µM

Compound	Fold induction (± SD)
Catechols	
*ȃ*Benserazide	1.0 ± 0.1
*ȃ*Dopamine	0.9 ± 0.1
*ȃ*Epigallocatechin gallate	1.0 ± 0.3
Quinones	
*ȃ*Menadione	0.0 ± 0.0
*ȃ*Thymoquinone	0.3 ± 0.0
Phenolic mannich bases and hydroxyphenylhydrazones	
*ȃ*Clofazimine	0.1 ± 0.0
*ȃ*Topotecan	0.9 ± 0.1
Natural products with other reactive groups	
*ȃ*Artemisinin	0.8 ± 0.1
*ȃ*Carfilzomib	0.7 ± 0.1
*ȃ*Mometasone furoate	0.6 ± 0.1
Natural products with nonspecific global interference properties	
*ȃ*Capsaicin	0.8 ± 0.0
*ȃ*Genistein	0.0 ± 0.0
*ȃ*Toxoflavin	0.0 ± 0.1

Data generated using Beta-Glo as the β-gal substrate.

### Initial evaluation of the P_murZ_ biosensor as a screening tool

Having established that the P*_murZ_* biosensor responds appropriately and uniquely to CWBIs, we next sought to validate its performance in a screening context, i.e. testing of multiple compounds in parallel, at a fixed compound concentration, with the number of processing steps reduced to an absolute minimum. For this purpose, we used several compound collections, including the NIH Clinical Collection (*n = *727), a subset of the MicroSource Spectrum library (*n *= 2000) and the Tocriscreen Total collection (*n = *1120), all of which include compounds in clinical use (or clinical trials) for a range of therapeutic indications, including antibacterial chemotherapy (*n = *156). To reduce processing steps in the assay, we replaced MUG with Beta-Glo reagent for quantifying β-gal production; this removed the need for centrifugation, wash and lysis steps in the original assay workflow, instead requiring only the addition of a single detection reagent direct to the challenged biosensor culture. The switch to Beta-Glo also dramatically improved the level of induction observed in the presence of CWBIs, with typical induction levels increasing from the single-digit values seen with MUG to >10 (data not shown). We took the opportunity of this improvement in signal-to-noise ratio to raise the induction threshold for this screen to ≥3-fold, thereby increasing discrimination in the assay.

In a screen of all 3847 compounds tested at a fixed concentration of 10 µM, the P*_murZ_* biosensor correctly identified 34 of the 46 CWBIs present, exhibiting ≥3-fold induction in all cases. Of the 12 CWBIs that failed to induce the biosensor, 3 were not detected in this initial screen because they had lost activity; resupply and re-test of these compounds confirmed that the P*_murZ_* biosensor is induced above the threshold in their presence. For the remaining 9 non-inducing CWBIs, we established that the chosen screening concentration of 10 µM was too far below the respective MIC of the compound to trigger induction. When these CWBIs were screened at a higher concentration (100 µM or 1000 µM), all 9 achieved ≥3-fold induction of the P*_murZ_* biosensor. A further 9 non-antimicrobial compounds also appeared to elicit a positive response from the biosensor on initial screening; however, resupply of these compounds and re-test at a range of concentrations established that they do not in fact cause induction (data not shown). Collectively, these results provide further confirmation of the high specificity and sensitivity of the P*_murZ_* biosensor, and in a context relevant to screening.

### Generation, validation and deployment of a P_murZ_ biosensor assay for high-throughput screening

Ahead of utilising the P*_murZ_* biosensor for a screen of natural products extracts to identify novel CWBIs, we first sought to further refine and validate the assay for such a purpose. We determined that the assay would accept miniaturization to a more high-throughput-friendly 384-well plate format (working volume of 50 µL/well), and could tolerate the presence of the extract solvent (DMSO) at typical working concentrations of up to 10% in this test format (data not shown). The performance of this optimized assay was assessed by confirming that the robust screening window coefficient (RZ′) was ≥0.5;^55,56^ utilising penicillin and vancomycin as positive controls, and running the assay in 10× 384-well plates on different days, the RZ′ was 0.65 and 0.64, respectively.

**Figure 2.**
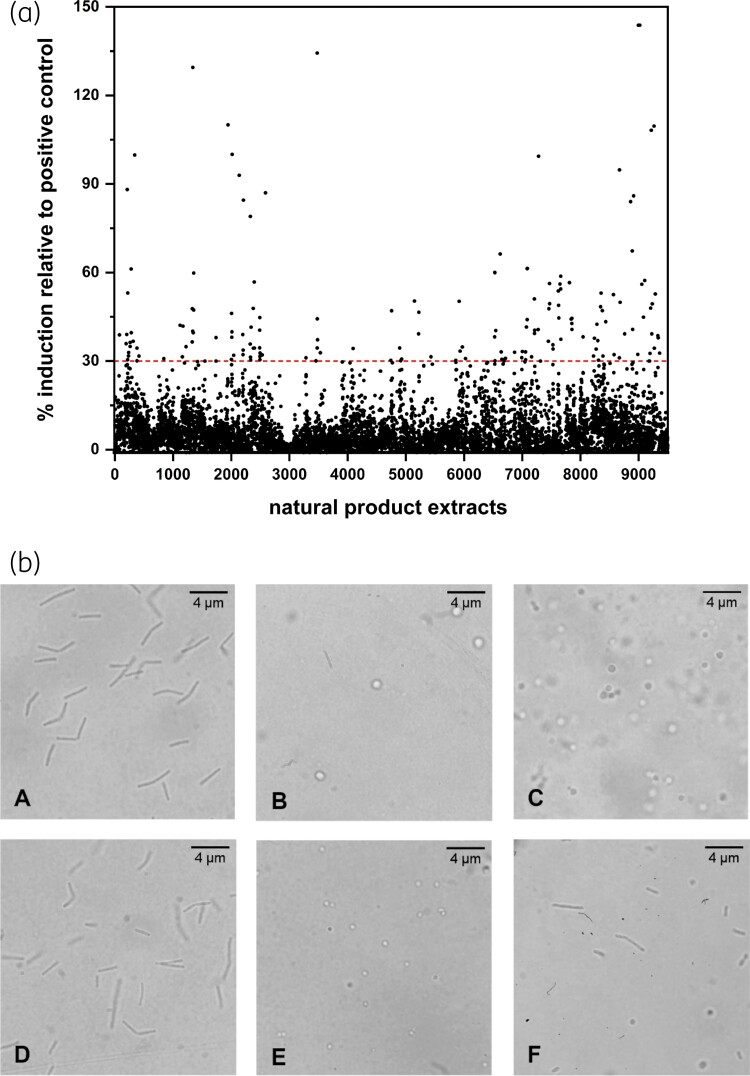
The P_murZ_ biosensor identifies a putative CWBI in a large-scale screen of natural product extracts. (a) Screening of 9328 extracts from the NCI Open Repository Program initially returned 165 hits that induced the biosensor at or above the threshold (30% of the positive control, indicated by a dashed line); of these, two were confirmed as inducers (i.e. potential CWBIs) upon re-test. (b) The two hits were further evaluated in the *B. subtilis* spheroplasting assay, which detects CWBIs by their ability to transform rod-shaped cells (A) into refractile spheroplasts. After 3 h, hit extract I (E) prompted spheroplast formation similar to that seen for known cell-wall active agents, penicillin (B) and lysozyme (C) (the latter both shown at 1 h). By contrast, hit extract II (F) did not induce spheroplast formation, akin to challenge with the non-CWBI antibiotic, tetracycline (D; shown after 3 h incubation). This figure appears in colour in the online version of *JAC* and in black and white in the print version of *JAC*.

We then employed the P*_murZ_* biosensor to screen a subset of the natural product extracts available through the NCI Open Repository Program. To mitigate the unworkably high hit rate (>6%) seen in this screen when using a fixed induction threshold of ≥3-fold, we instead calculated a threshold value per screening plate, setting it at ≥30% of the positive control (10 µM penicillin) induction value for that plate. From a total of 9328 extracts screened, 165 induced the biosensor above the latter threshold [Figure 2(a)], an overall hit rate of ∼1.8%. Subsequent retest of these hit extracts with the P*_murZ_* biosensor returned only six hits. Of these, only two (hereafter referred to as hit extracts I and II) still showed induction when testing resupplied extract (data not shown).

We attribute the poor reproducibility in this screen to sample degradation upon storage, rather than reflecting a fundamental issue with biosensor performance. Extracts were stored for extended periods in solution at −20°C between primary screen and re-test, and experienced freeze–thaw during consolidation of hit extracts to plates for re-test. The idea that sample degradation occurred under these conditions is supported by the observation that the induction values for extracts upon re-test were considerably lower than those seen in the primary screen; the mean fold induction value across all 165 hits in the screen was 11.3, whilst for the 6 hits from the re-test, mean fold induction was 3.9.

### Further characterisation of hit extracts apparently containing a CWBI

Two approaches were taken to further corroborate the presence of CWBIs in the two hit extracts. First, we assessed the response of another CWBI biosensor (P*_sgtB_*) to the extracts, in both cases observing induction above the threshold (≥30% of the positive control) (data not shown), and thereby reinforcing the idea that these extracts contain a CWBI. Second, we evaluated the ability of the extracts to induce spheroplasting in *B. subtilis*; during growth in osmotically buffered media, challenge with a CWBI will transform the rod-shaped bacilli into refractile spherical cells that are readily detectable by microscopy.^2^ Hit extract I induced spheroplast formation [Figure 2(b)], providing further orthogonal confirmation for the presence of a CWBI in this case and underscoring the utility of the P*_murZ_* biosensor as a screening tool to detect CWBIs. Hit extract II did not cause spheroplasting [Figure 2(b)], and may therefore not contain a CWBI. However, further analysis will be required to confirm the latter result, since the spheroplasting assay can suffer from false-negative results when using extracts that contain a CWBI and another antibacterial compound (e.g. a translation inhibitor that blocks the necessary growth for spheroplast formation, or a membrane-active agent that triggers spheroplast lysis).^4^

We next sought to assess whether hit extract I contained a known or a novel CWBI. The extract in question is of fungal origin, and fungi are known to produce several CWBIs that include small-molecule antibiotics such as the β-lactams and fosfonochlorin,^57^ and peptide defensins like plectasin,^58^ eurocin^59^ and copsin.^60^ Tandem MS analysis of hit extract I yielded no spectral matches corresponding to known CWBIs or antibiotics in GNPS,^61^ and no defensin-like peptides were detected using PEAKS DB (data not shown).^62^ Thus, the CWBI in extract I may be novel, though further work will be required to confirm this conclusion and to establish the compound’s identity.

### Conclusions

The *S. aureus* P*_murZ_* reporter strain is—to our knowledge—the first extensively validated biosensor demonstrated to respond appropriately and uniquely to CWBIs. Crucially, it does not exhibit the non-specific induction by membrane-active agents generally observed with existing CWBI biosensors, making it a particularly powerful tool for screening in contexts (e.g. the typical synthetic compound library) where nuisance compounds of this type are commonplace. Further underscoring its utility for antibacterial discovery, we have shown that this biosensor can be deployed in a robust, high-throughput assay that successfully identifies candidate CWBIs, even in the context of complex natural product extracts. In addition to its value in primary screening, the high specificity and sensitivity of the P*_murZ_* biosensor make it a valuable tool to interrogate the antibacterial mode of action of compounds initially discovered through other means.

## Supplementary Material

dkac429_Supplementary_Data

## Supplementary data

Tables S1 to S3 are available as Supplementary data at *JAC* Online.
