# Supplementary material for: An extensively validated whole-cell biosensor for specific, sensitive and high-throughput detection of antibacterial inhibitors targeting cell-wall biosynthesis
Source: J Antimicrob Chemother. 2023 Jan 10;78(3):646–55. doi: 10.1093/jac/dkac429 (PMC9978594; doi:10.1093/jac/dkac429)
Supplement: dkac429_Supplementary_Data [file dkac429_supplementary_data.docx]

**Table S1. Oligonucleotide primers used in this study.**

| **Designation** | **Sequence (5’-3’)** | **Application** |
| --- | --- | --- |
| gltB-U | TTCTGGTACCTTAGGGATGACATGTGTATC | Amplification of region upstream of *gltB* |
| gltB-L | ATGCAGGATCCTTTTTTAGAATATTCTGAT |  |
| ilvD-U | AATGGTACCAACGTTGTTTTACCTGCTCCT | Amplification of region upstream of *ilvD* |
| ilvD-L | CCGGATCCAATTTTAATGATTAATCATGTT |  |
| lacZ-U | ATGGGATCCTATAGGGAAAAGGTGGTGAACTACTG | Amplification of *lacZ* from pMUTIN4 |
| lacZ-L | TTAGAAGCTTATAGTACATAATGGATTTCCTTACG |  |
| oppB-U | TTTCGGTACCCTAAGATGAGCGACAGCACA | Amplification of region upstream of *oppB* |
| oppB-L | ATCTAATGGATCCCTCTAACAAATTCCGAT |  |
| sgtB-U | TCGAGGTACCCATTGCAGAAGCTAAACCAG | Amplification of region upstream of *sgtB* |
| sgtB-L | GTTTGGGATCCCTTTTAAAACTCACTTAGT |  |
| 2768-U | TTGGGTACCCACCTACAACTTTGCTTTCTG | Amplification of region upstream of *ORF2768* |
| 2768-L | GCGCGGATCCTAAGTCCAAAATCTGATTGC |  |

**Table S2. Additional sources of chemical compounds used in this study.** Compounds in bold were received as gifts.

| **Compound** | **Source** |
| --- | --- |
| actinonin, artemisin, benserazide, carfilzomib, genistein, topotecan, mometasone furoate | Insight Biotechnology (Wembley, UK) |
| aztreonam | MP Biochemicals (Illkirch, FR) |
| β-chloro-D-alanine | Toronto Research Chemicals (Toronto, Ontario, CA) |
| cefalexin, vancomycin | Cayman Chemical (Ann Arbor, Michigan, USA) |
| cloxacillin, gepotidacin, rifampicin | Cambridge Bioscience (Cambridge, UK) |
| EGCG | Bio-Techne (Abingdon, UK) |
| flucloxacillin | CP Pharmaceuticals (Wrexham, UK) |
| linezolid | ChemCruz (Heidelberg, DE) |
| mecillinam | TOKU-E (Washington, USA) |
| telavancin | AdooQ Bioscience (Irvine, California, USA) |
| **daptomycin** | Cubist Pharmaceuticals (Lexington, Massachusetts, USA) |
| **deoxyactagardine B, mersacidin** | Novacta Biosystems (Hertfordshire, UK) |
| **friulimicin** | Tanja Schneider (University of Bonn; Bonn, DE) |
| **murgocil** | Terry Roemer (Merck; Kenilworth, New Jersey, USA) |

**Table S3. Three *S. aureus* biosensor constructs that exhibit induction of β-gal** **expression at or above the threshold (green) specifically in response to cell-wall active agents.** Data generated using MUG as the β-gal substrate, with the induction threshold set at 2-fold.

| **Antibacterial agent** | **Fold induction (± standard deviation)** | | | | | | | | |
| --- | --- | --- | --- | --- | --- | --- | --- | --- | --- |
|  | **P*_gltB_*** | | | **P*_sgtB_*** | | | **P*_murZ_*** | | |
|  | **0.25XMIC** | **1XMIC** | **4XMIC** | **0.25XMIC** | **1XMIC** | **4XMIC** | **0.25XMIC** | **1XMIC** | **4XMIC** |
| ***inhibitors targeting the cell-wall*** | | | | | | | | | |
| fosfomycin | 0.8 ± 0.1 | 0.9 ± 0.2 | 2.4 ± 0.2 | 1.3 ± 0.3 | 2.1 ± 0.4 | 4.8 ± 0.6 | 1.9 ± 0.1 | 3.1 ± 0.4 | 4.6 ± 0.9 |
| bacitracin | 3.0 ± 0.3 | 2.5 ± 0.3 | 2.6 ± 0.2 | 5.4 ± 0.7 | 4.7 ± 0.6 | 3.8 ± 0.8 | 5.5 ± 0.4 | 6.7 ± 0.7 | 5.3 ± 0.3 |
| vancomycin | 1.9 ± 0.5 | 3.2 ± 1.5 | 2.0 ± 0.1 | 1.1 ± 0.2 | 4.0 ± 0.3 | 5.0 ± 1.5 | 0.7 ± 0.1 | 3.7 ± 0.4 | 3.8 ± 0.5 |
| methicillin | 1.0 ± 0.0 | 1.5 ± 0.0 | 4.9 ± 0.4 | 1.0 ± 0.0 | 2.6 ± 0.2 | 2.4 ± 0.1 | 1.4 ± 0.0 | 4.6 ± 0.1 | 10.4 ± 0.1 |
| mersacidin | 2.7 ± 0.2 | 2.8 ± 0.5 | 2.0 ± 0.1 | 2.0 ± 0.4 | 2.5 ± 0.1 | 2.8 ± 0.5 | 2.4 ± 0.3 | 3.7 ± 0.8 | 2.9 ± 0.3 |
| ***inhibitors of other targets*** | | | | | | | | | |
| polymyxin B | 0.8 ± 0.1 | 1.1 ± 0.5 | 0.9 ± 0.1 | 1.0 ± 0.1 | 1.1 ± 0.1 | 1.5 ± 0.6 | 1.2 ± 0.2 | 1.2 ± 0.1 | 1.3 ± 0.0 |
| novobiocin | 1.1 ± 0.4 | 1.0 ± 0.3 | 1.1 ± 0.3 | 1.0 ± 0.2 | 1.0 ± 0.2 | 1.0 ± 0.1 | 1.3 ± 0.4 | 1.2 ± 0.4 | 1.8 ± 0.1 |
| rifampicin | 0.9 ± 0.2 | 1.2 ± 0.2 | 0.8 ± 0.3 | 0.4 ± 0.1 | 1.1 ± 0.5 | 1.1 ± 0.2 | 0.7 ± 0.3 | 0.5 ± 0.1 | 0.4 ± 0.1 |
| gentamicin | 1.0 ± 0.2 | 0.9 ± 0.1 | 0.9 ± 0.1 | 0.7 ± 0.1 | 0.6 ± 0.1 | 1.4 ± 0.4 | 1.1 ± 0.2 | 1.1 ± 0.1 | 1.0 ± 0.1 |
| triclosan | 1.0 ± 0.1 | 0.4 ± 0.1 | 1.4 ± 0.2 | 1.2 ± 0.2 | 1.2 ± 0.1 | 0.8 ± 0.1 | 0.5 ± 0.1 | 0.4 ± 0.2 | 0.9 ± 0.1 |
